# Supplementary material for: Measurement of the Mapping between Intracranial EEG and fMRI Recordings in the Human Brain
Source: Bioengineering (Basel). 2024 Feb 27;11(3):224. doi: 10.3390/bioengineering11030224 (PMC10968112; doi:10.3390/bioengineering11030224)
Supplement: Supplementary file 1 [file bioengineering-11-00224-s001.zip › bioengineering-2691723-supplementary.pdf]

bioengineering MDPI

Supplementary Material

Simulation of 'heuristic' measures: methods

To better characterise and illustrate the behaviour of the metrics in the Appendix we performed simulations of different patterns of spectral fluctuations. Discrete spectra were created with 1024 points within a range of 1–129 Hz. The power at each frequency was determined by the addition of a 1/f distribution and two Gaussian peaks were added to this spectrum at a 'low' 10 Hz and 'high' 60 Hz frequency with full width at half maximum of 4 Hz. In order to simulate dynamic processes, we first increased the amplitude of the power at these frequencies up to a 100% of the power at that frequency in the 1/f spectrum (see Figure S1). Secondly, each peak was shifted in frequency upwards in steps of ~0.1 Hz from 0–8 Hz. The expected BOLD response was then determined using the range of 'heuristic' measures derived in the Appendix.

Results

The simulated spectra containing changes in peak *amplitude* are illustrated in figure S1. The simulated spectra containing changes in peak *frequency* are illustrated in Figure S2. The corresponding predicted BOLD changes generated by the different 'heuristic' measures are shown in Figure S3. For changes in peak amplitude (and therefore power) at a given frequency (top row of Figure S3), the q_{RMSF} model predicts BOLD increases associated with increased power at high frequencies, and decreases with increased low frequency power. This is in contrast the $q_{MSF'}$, which predicts BOLD increases with either increased low or high frequency power, although the predicted BOLD increase is greater at higher frequencies. The CofM metric is similar to the q_{RMSF} metric in its behaviour, with the distinction that the ICofM predicts an equal change in BOLD for the same percentage change at 10 Hz or 60 Hz. The I_{40Hz} metric predicts BOLD increases with increased power at 10 or 60 Hz, with a greater effect at 10 Hz than 60 Hz. For the heuristic metric q_{RMSF} (13) frequency shifts in peaks at low and high frequencies predict a BOLD increase, with a greater effect at lower frequencies. The $q_{MSF'}$ metric similarly shows a BOLD increase for a shift to higher frequencies of either a low or high frequency peak with a greater effect at high frequency.

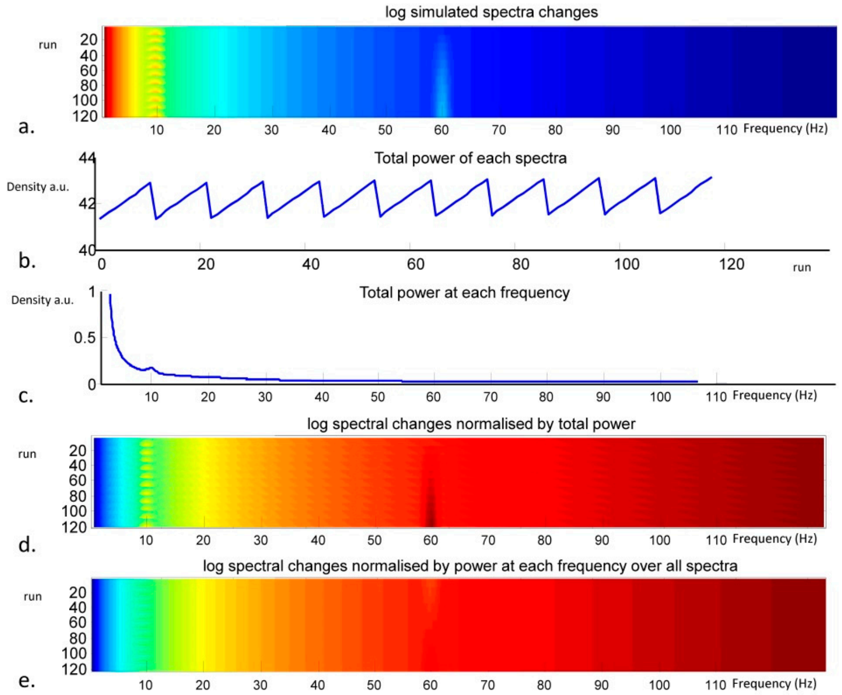

Figure S1. Simulated spectra varying peak amplitude.

Spectra were simulated as a 1/f spectrum with two peaks at 10 and 60 Hz. In each spectra the amplitude was altered (a). The variability in the total power for each simulated spectrum is shown (b). The average spectrum is calculated (c). The effect of normalising by the total power is shown in (d) and normalising by the average power at each frequency (e).

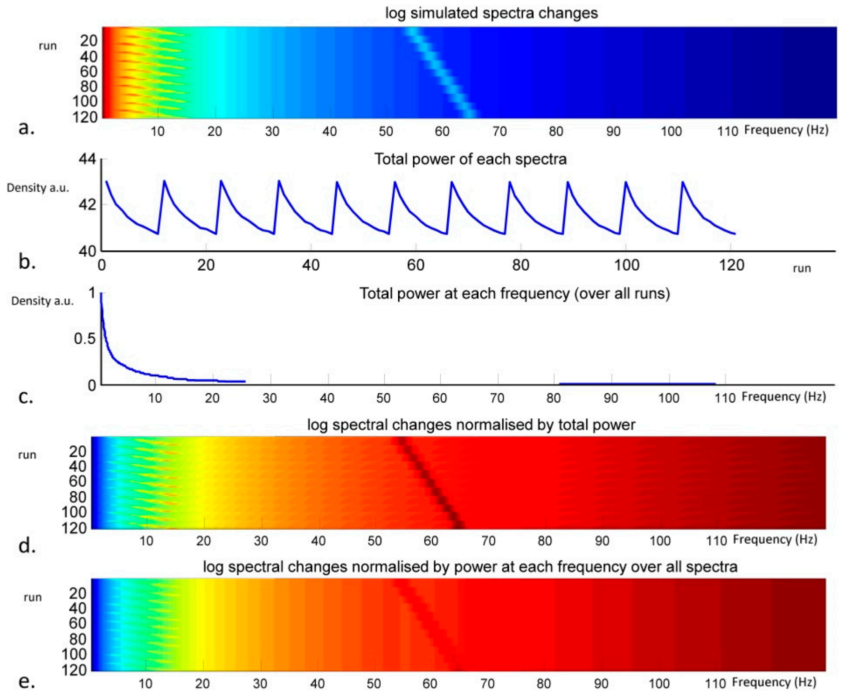

Figure S2. Simulated spectra varying peak frequency.

Spectra were simulated as a 1/f spectrum with two peaks at 10 and 60 Hz. In each spectra the peak frequency was altered (a). The variability in the total power for each simulated spectrum is shown (b). The average spectrum is calculated (c). The effect of normalising by the total power is shown in (d) and normalising by the average power at each frequency (e).

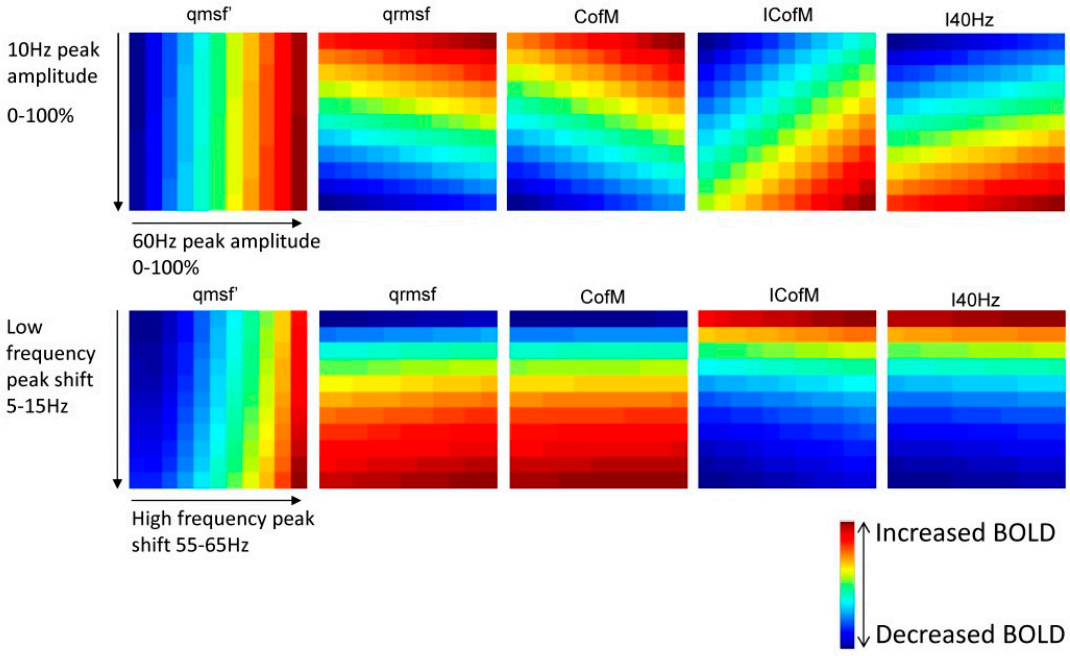

Figure S3. Simulated BOLD changes predict by different cross-spectral metrics.

The predicted responses by each of the cross spectral predictors defined in appendix 1 to the simulated spectral changes are shown. The top row shows the predicted BOLD response to changes in peak amplitude (spectra in Figure S1) for each metric and the bottom row the BOLD response predicted to changes in peak frequency (spectra in Figure S2).
